# Immunology and immunotherapy in breast cancer

**DOI:** 10.20892/j.issn.2095-3941.2021.0597

**Published:** 2022-06-09

**Authors:** Vladimir Semiglazov, Andrey Tseluiko, Asel Kudaybergenova, Anna Artemyeva, Petr Krivorotko, Roman Donskih

**Affiliations:** 1 Petrov National Medicine Cancer-Research Center Ministry of Health, Saint-Petersburg 197758, Russia

**Keywords:** Breast cancer, tumor-infiltrating lymphocytes, PD-1, PD-L1, immunotherapy

## Abstract

Immuno-oncology is a rapidly developing field in medicine. Drug combination therapies have already been studied in many clinical trials on various tumor types. In recent years, a checkpoint inhibition therapy with monoclonal antibodies targeting PD-1 and its ligand PD-L1 has been developed. Breast cancer had been examined in the field of immune-oncology relatively recently. This review focuses on clinical evidence regarding immune checkpoint inhibition for curative treatment of various breast cancer subtypes. In addition, we present the results of studies demonstrating the prognostic and predictive value of levels of tumor-infiltrating lymphocytes (CD4 and CD8), their quantitative ratios, and their correlation with regulatory genes (PD-1, PD-L1, and FOX-P3).

## Introduction

Breast cancer (BC) is a complex disease whose biology, morphology, and clinical history vary. BC comprises heterogeneous subtypes with distinct biology, morphology, and prognosis.

The immune system plays a dual role in BC development and progression, which is best explained by immuno-editing, in which tumors are subjected to selective immune pressures that promote immune-editing, and ultimately immune escape. A better understanding of early events in tumor growth and progression should guide the development of effective immunotherapies that promote a shift toward tumor elimination^1,2^.

The selection of systemic therapy for BC has traditionally been based on the expression of estrogen receptor (ER), progesterone receptor (PR), and/or human epidermal growth factor receptor 2 (HER2). According to the expression of these biomarkers, BC is divided into 4 intrinsic subtypes: luminal (A and B), HER2^+^, and triple-negative BC (TNBC). These subtypes in most cases have specific immunological characteristics, differing in the quantity of tumor-infiltrating lymphocytes (TILs), programmed death ligand 1 (PD-L1) expression, and tumor-associated antigens, as well as the tumor mutational burden^3–7^.

Notably, the investigation of immunotherapy initially proceeded more slowly for BC than other solid tumors, although trials are increasingly investigating immunotherapeutic agents^7^. These trials are being conducted in metastatic and early settings in parallel; this study design is interesting given that modern drugs are most often studied in patients with advanced stages of the disease, and only then used for early stages^3^.

## Immunosurveillance of BC

### BC is immunogenetic

**Figure 1:**
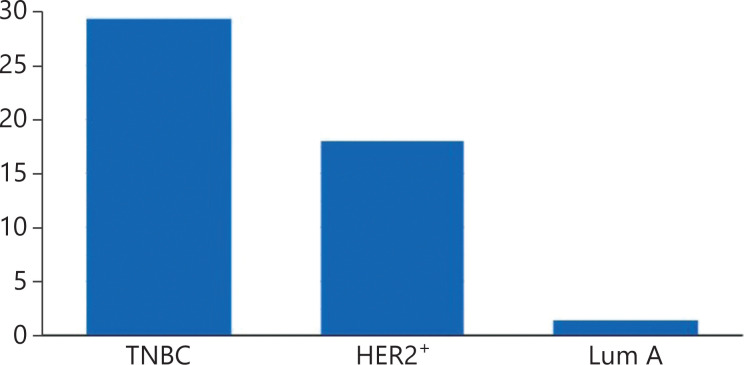
PD-L1 positivity in different BC subtypes.

BC has historically been viewed as immunologically silent. However, some subtypes of breast cancer naturally induce an adaptive immune response, contain tumor-infiltrating T-cells at diagnosis, and also express PD-L1^7^. Both TIL content and PD-L1 expression vary in the major clinical subtypes (**Figure 1**) of BC^6,8^. Lymphocyte predominant BCs are breast tumors with stromal or intratumoral lymphocytes that account for more than 50%–60% of the tumor tissue^9^, although a linear rather than a dichotomous relationship exists between the TIL content of BCs and clinical outcomes. The presence of TILs at diagnosis confers both prognostic and predictive information, regardless of BC subtype^10–15^.

### TILs have prognostic value

A retrospective-prospective analysis of almost 16,000 patients in 5 different trials has demonstrated that the presence of stromal TILs at diagnosis is prognostic for TNBC and HER2^+^ BC, but not for luminal ER^+^ BC^10^. In 821 patients with TNBC across 3 trials, an approximately 15%–20% improvement in disease-free survival (DFS) for each 10% increase in stromal TILs has been observed through hematoxylin and eosin (H&E) staining (*P* = 0.005 to 0.035). In 387 patients with HER2^+^ BC, the rate of recurrence decreased 3% for every 1% increase in stromal TILs, according to H&E staining (*P* = 0.002)^11^. Another study has evaluated 12,366 patients for either stromal or intratumoral CD8^+^ TILs and demonstrated no influence of intratumoral CD8^+^ TILs on BC-specific survival for ER^+^ BC, but an effect of stromal CD8^+^ TILs on survival for ER^-^HER2^+^ BCs^15^.

### TILs are predictive of therapy response

TILs may also be a predictive biomarker of the response to therapy. Elevated levels of TILs at diagnosis are associated with greater benefit from adjuvant anthracycline therapy^16^, in agreement with data indicating that anthracycline based chemotherapy induces immunogenic cell death *via* the Toll-like receptor 4 (TLR-4) pathway.

Data on adjuvant trastuzumab-based therapy have been conflicting. In the FinHER study, greater TIL density has been associated with the response to trastuzumab^17^, whereas no such association has been found in the NCCTG N9831 study^18^. Moreover, stromal TILs (on the basis of H&E staining) can predict cPR to neoadjuvant chemotherapy. The rate of cPR in all BC subtypes is approximately 30% higher when breast tumors contain >60% TILs rather than ≤60% TILs^10,11^. Adding carboplatin to standard neoadjuvant therapy increases the cPR rates of HER2^+^ BCs from 50% to 72% and TNBCs from 43% to 74%, respectively (*P* < 0.005)^19^. Residual disease after neoadjuvant chemotherapy is a poor prognostic factor for HER2^+^ BC and TNBC^20^, but patients with TILs in their residual tumors have better prognosis.

## TILs in BC subtypes

The availability of TILs is an important prognostic factor in BC, and TILs may synergize with chemotherapy and immune checkpoint inhibitor therapy in eliciting a clinical response (CR). Understanding of variations in lymphocytic infiltration in BC may help support the identification of subtypes more amenable to immunomodulation.

Determining the median percentage of patients with absent TILs, intermediate TILs, or high levels of TIL, and assessing the variations in lymphocytic cell subsets in different BC subtypes, is crucial. Stanton et al.^5^ and Urezkova et al.^21^ have analyzed studies of tumor lymphocytic, CD8^+^, and FOXP3^+^ cellular infiltrates, and used multivariable analyses and quantitative methods to enumerate cell populations. Study selection was performed in accordance with the PRISMA guidelines and evaluated by 2 independent appraisers.

A median of 11% (range, 5%–26%) of BCs were LPBC (Lymphocyte-predominant breast cancer), and approximately 16% of cancers showed no evidence of TILs. TNBCs showed the highest incidence of LPBC (20%; range, 4%–37%). This incidence is similar to that observed in HER2-positive BCs (16%; range, 11%–24%). Hormone receptor positive (HR^+^)/Her2neu negative (HER2^-^) BCs showed a lower incidence of LPBC, at 6% (range, 3%–12%). CD8^+^ T-cell infiltrates, indicative of type I immunity, were found in 48% of all BCs (range, 32%–80%), and similar levels were observed in TNBC (60%; range, 40%–91%) and HER2^+^ disease (61%; range, 40%–83%). Fewer HR^+^ tumors showed CD8^+^ TILs (43%; range, 30%–73%). The highest levels of forkhead box protein 3 (FOXP3) cells were observed in TN (70%; range, 65%–76%) and HER2^+^ disease (67%; range, 61%–74%). A minority of HR^+^ BCs showed high levels of tumor-infiltrating FOXP3^+^ cells (38%; range, 35%–41%).

The prevalence (presence) of TILs varies within and between subtypes of BC. Levels of lymphocytic subpopulations may recognize BCs that are more amenable to immunomodulation and indicate additional strategies to enhance immunity in patients with low to moderate levels of TILs.

### TNBC

Three times as many TNBCs are classified as LPBC than HR^+^ disease cases. Many factors may promote to the value of adaptive immunity in TNBCs^22^. First, HR^-^ cancers have been shown to have higher genomic and chromosomal instability than HR^+^ BCs^23,24^. However, whereas luminal A (HR^+^ BC) tumors have low genomic instability, some luminal B HR^+^ tumors have high genomic instability^25–27^. A large number of mutations increases the chance of mutated protein sequences being expressed and potentially recognized as novel antigens by the immune system, thereby inducing an immune response. TNBC is also associated with many aberrant signaling pathways, such as EGFR, MET, and PI3K^28^, in which multiple phosphorylated proteins increase the expression of various phosphopeptides that may also be recognized as foreign by the immune system and elicit an immune response^29^. However, TNBCs have been reported to be infiltrated with B-cells. A meta-gene signature including B-cells has been associated with improved survival in basal TNBC^30^. Few investigations have fully characterized TILs for levels of T *vs* B lymphocytes. More specific delineation of T-cells and B-cells within immune infiltrates might improve identification of the role of each lymphocyte type in BC prognosis.

### HER2^+^ BC

Patients with HER2^+^ BC have similar LPBC to those with TNBC and usually show infiltration with CD8^+^ T-cells. The presence of LPBC and CD8^+^ TILs is not predictive of prognostic benefits in HER2^+^ BC, as in TN BC^17,31,32^. One possible explanation is that the immune infiltrate in the HER2^+^ subtype must be considered in the context of hormonal status. One study has demonstrated that CD8^+^ infiltrate in HR^-^/HER2^+^ tumors, but not HR^+^/HER2^+^ tumors, is predictive of improved RFS (*P* = 0.04), thus suggesting that the immune-mediated prognostic benefit is due to the negative HR status rather than HER2^+^ overexpression^8,33^.

### HR^+^ BC

Responses to immunotherapy are uncommon in ER^+^ BC and lack predictive markers to date. A role of the immune response in HR^+^ disease taken into consideration, because high levels of FOXP3^+^ TILs predict poor prognosis in HR^+^ tumors^34^. The roles of regulatory T-cells (Tregs) in suppressing functional T-cells in other subtypes is unclear. Most published studies have evaluated Tregs by using only a single marker and have not stratified for FOXP3 expression in CD4^+^ or CD8^+^ T-cells. These aspects are important because FOXP3 is a marker expressed on activated CD8^+^ T-cells^35^. High levels of FOXP3^+^ TILs are associated with poor survival in HR^+^ BCs that lack CD8^+^ TILs but not in other BC subtypes in which Tregs may include activated CD8^+^ lymphocytes^36^. Moreover, tumor-infiltrating follicular CD4^+^ T-cells near CD8^+^ T-cells have been associated with better prognosis in HR^+^ BC^37^. Data suggest that evaluation of both intratumoral CD4^+^ and CD8^+^ T-cells may be needed to fully assess the immune environment of the HR^+^ subtype^35–37^. Targeting both Treg and Erα may reverse the immunosuppressive environment of HR^+^ BC, thereby potentially enabling the effectiveness of immune-modulating therapies in HR^+^ BC.

HR^+^BCs, compared with other subtypes, are associated with lower levels of TILs, tumor mutational burden, and PDL-1 expression.

Regarding metastatic cancer outcomes, the combination of pembrolizumab with eribulin, compared with eribulin alone, has demonstrated no additional benefits in terms of PFS, OS, or overall response rates. Importantly, in this trial, 61% of the patients had received prior chemotherapy for metastatic disease, and 73% had received prior treatment with CDK4/6 inhibitors, which might have contributed to these results^38^.

In contrast, in the I-SPY2 study, the combination of pembrolizumab with neoadjuvant chemotherapy almost tripled the pathologic complete response (pCR) rates for early HR^+^ BC, thus suggesting that, similar to observations in in TNBC, efficacy of immunotherapy may be better in early-stage breast cancer^39–41^.

Some data have defined a novel immune signature in patients with PD-L1^-^ ER^+^ BC, who are likely to benefit from immune-checkpoint and histone deacetylase inhibition (NCT02395627)^42^.

Histone deacetylase inhibitors (HDACi), such as vorinostat, are epigenetic modifiers that reverse hormone therapy resistance, thus prolonging anti-tumor responses in patients.

Beyond their effects on ER signaling, HDACi have been suggested in preclinical studies to decrease Tregs, induce PD-L1 expression on tumor cells, and alter the composition of TILs, specifically inducing CD8^+^ T-cells *in vitro* and *in vivo* in BC models^42^.

## Peritumoral and intratumoral FOXP3^+^ Tregs in patients with BC

Liu et al.^43^ have reported that the prognostic value of tumor-infiltrating FOXP3 Tregs in breast carcinoma depends on their relative density and tissue locations. Liu et al.^43^ have assessed the changes in Tregs before and after neoadjuvant chemotherapy, and assessed their relationships with tumor response and patient survival. The changes were significant in tumors that usually respond to neoadjuvant chemotherapy, including the HER2-enriched and basal-like subtypes (*P* = 0.035; *P* = 0.004). Univariate and multivariate analyses indicated that decreased peritumoral Tregs are an independent predictor of pCR, and the intratumoral Tregs after chemotherapy are associated with overall survival and progression-free survival in patients. Peritumoral Tregs are sensitive to chemotherapy and associated with pCR, whereas intratumoral Tregs are an independent prognostic predictor in patients with BC.

Miyashita et al.^44^ have studied the prognostic value of CD8^+^ TILs and FOXP3^+^ TILs in residual tumors after neoadjuvant chemotherapy (NAC), and the alterations in these parameters before and after NAC in patients with TNBC.

Subclassification of TILs is crucial; for instance, some studies have reported that cytotoxic (CD8^+^) T-cells are associated with improved clinical outcomes in patients with BC^15,32,45,46^, whereas other studies have not confirmed this association^39^. In addition, Tregs defined as FOXP3^+^ T-cells play a crucial role in suppressing antitumor immunity^15,32^. However, the prognostic roles of FOXP3 remain controversial; for instance, BCs with FOXP3^+^ TILs have been reported to be less sensitive to cytotoxic chemotherapy and to have a poorer prognosis in some studies^39,46^, whereas other studies have reported that BCs with FOXP3^+^ TILs have a better prognosis^47,48^.

Recent preclinical studies have revealed that cytotoxic agents may exert antitumor activity by inducing an immune response against tumor cells^49^.

Miyashita et al.^44^ have reported 5-year RFS rates of 72% and 40% in patients with high and low CD8/FOXP3 ratios, respectively, and 5-year BCSS rates of 77% and 56% in patients with high and low CD8/FOXP3 ratios, respectively.

Miyashita et al.^44^ have also demonstrated that high CD8^+^ TIL levels and CD8/FOXP3 ratios in residual tumors accurately predict better clinical outcomes in patients with TNBC with non-pCR after NAC, and that the changes in these parameters in BC tissues after NAC are significantly associated with eventual clinical outcomes in TNBCs. These parameters may serve as a substitute for adjuvant treatment in patients with residual disease in the neoadjuvant setting.

The presence of FOXP3 expression in ER^+^ BCs on intratumoral lymphocytes is significantly associated with a trend toward lower overall survival rate (*P* = 0.06). An analysis of the literature has also shown that FOXP3 is a marker of a poor prognosis, particularly in ER^+^ carcinoma, but a favorable prognosis in HER2^+^/ER^-^ carcinoma^37^.

## Immunotherapy of advanced and metastatic TNBC

Most data investigating chemo-immunotherapy approaches have been generated in TNBC. Some reasons why research has focused on this subtype are that TNBC is characterized by a more robust immune infiltrate, higher levels of PD-L1 expression, and the presence of genomic instability, with a higher level of non-synonymous mutations, than other BC subtypes^50–53^.

TNBC describes BCs that lack ER and PR expression and do not overexpress HER2. Patients with TNBC have poor clinical outcomes. Chemotherapy remains the primary systemic treatment, and international guidelines support the use of single-agent taxanes or anthracyclines as a first-line therapy. Estimates of the median overall survival vary but remain at approximately 18 months or less. In patients with TNBC, the expression of PD-L1 occurs mainly on tumor-infiltrating immune cells rather than on tumor cells^54^, and can inhibit anticancer immune responses^55,56^. Thus, the inhibition of programmed death 1 (PD-1) and PD-L1 may be a useful treatment strategy. Atezolizumab selectively targets PD-L1, thus preventing interaction with the receptors PD-1 and B7-1 (a costimulatory cell-surface protein), and reversing T-cell suppression^56^.

Unexpectedly, 2 of the the most important phase III randomized studies in metastatic or unresectable locally advanced TNBC (IMpassion130 and IMpassion131 trials) have used a similar design but indicated different survival results.

In the IMpassion130 trial, patients were randomized to nab-paclitaxel with either atezolizumab or placebo. As demonstrated previously^56–58^, PFS was statistically significantly prolonged in both the intention-to-treat (ITT) population and the PD-L1 positive population (≥1% expression on immune cells in the tumor area) in the atezolizumab arm. As presented in 2021^59^, the final OS analysis showed no statistically significant difference between arms in the ITT population. The trial had a hierarchical design for OS; therefore, OS in the PD-L1 positive population was not formally tested. However, an exploratory analysis in the PD-L1 IC positive population revealed a 7.5-month survival benefit with the addition of atezolizumab^57^.

The other international trial, IMpassion 131, evaluated atezolizumab in combination with paclitaxel *vs.* placebo plus paclitaxel in patients with metastatic TNBC^60^. Of the 651 patients, 45% had PD-L1 positive TNBC. At the primary endpoint PFS analysis, addition of atezolizumab to paclitaxel did not result in a statistically significant improvement in investigator-assessed PFS in the PD-L1 positive population. At the final data cut-off, deaths had been recorded for 123 (42%) of 292 patients in the PD-L1 positive population (44% *vs.* 39%) in the atezolizumab *vs* placebo arms, respectively). In the final analysis, grade 3/4 adverse events had occurred in 53% of atezolizumab-treated and 46% of placebo-treated patients^60^.

The findings from Impassion131 also contrast with recently published results from the KEYNOTE-355 trial, which has evaluated a broader range of chemotherapy backbones (including both nab-paclitaxel and paclitaxel, as well as gemcitabine/carboplatin) with a different immunotherapy agent, pembrolizumab^61^. The overall aim of KEYNOTE-355 was broadly similar to that of Impassion131, but important differences existed with respect to eligibility, PD-L1 testing, chemotherapy backbone, and statistical design. The RFS HR in the ITT population was 0.82, a value similar to that in Impassion131. However, the PFS HR in the PD-L1 positive population, although identified with a different assay, was 0.65. Despite a longer follow-up, the OS results have not yet been reported from KEYNOTE-355. Interestingly, no evidence has indicated that paclitaxel is a poorer chemotherapy partner than nab-paclitaxel, although the taxane backbone was chosen by the investigators, and therefore the populations treated with each formulation of paclitaxel may differ substantially.

The final OS exploratory analysis has suggested an effect of atezolizumab, with maintained and even enhanced separation of the curves. In IMpassion 131, no difference was observed between treatment arms during the first 7–8 months of treatment. The subsequent diversion of the curves prompts the question of why the difference between treatment arms occurred much later in Impassion 131 than Impassion 130. The influence of concomitant steroids during paclitaxel therapy might potentially have dampened the effect of immunotherapy. However, in the IMpassion131 trial, steroids were mainly used to prevent hypersensitivity reactions^62^. Thus, on the basis of the efficacy data of both KEYNOTE-355^61^ and KEYNOTE-522^63^, the difference in steroids alone cannot explain the distinct results between IMpassion131 and IMpassion130^64,65^.

## Role of immunotherapy in early TNBC

In the NeoTRIP trial, the addition of atezolizumab to neoadjuvant chemotherapy with carboplatin and nab-paclitaxel for 8 cycles led to a non-statistically significant increase of 4.2% of the pCR rate in women with high risk TNBC^66^. The rate of pCR was higher for tumors expressing PD-L1 than for PD-L1 negative cases.

Other trials have reported results on the addition of immune checkpoint inhibitors (ICIs) to neoadjuvant chemotherapy in women with high risk TNBC^67^. In Impassion031, atezolizumab increased pCR from 41% to 58% when added to sequential chemotherapy with nab-paclitaxel for 12 weeks, followed by doxorubicin and cyclophosphamide for 5 cycles^53^. Of interest, in that trial, the improved pCR rate with atezolizumab was independent of tumor PD-L1 expression—a finding different from the observation in NeoTRIP, and at odds with the results in women with metastatic TNBC, in whom a benefit from atezolizumab with nab-paclitaxel was restricted to PD-L1 + tumors^56^. In the KEYNOTE522 study of neoadjuvant docetaxel followed by an anthracycline regimen, the addition of PD-1 directed pembrolizumab was associated with a significantly higher (13.8%) rate of pCR in the planned analysis of the first 602 patients , although the difference in favor of pembrolizumab decreased to 7.4% when all patients enrolled into trials were considered. No data are available regarding potentially different sensitivity to pembrolizumab on the basis of PD-L1 expression, assessed with different reagents and a different scoring system than those for atezolizumab^63^.

The non-significant difference in pCR between arms in NeoTRIP requires further consideration. The neoadjuvant studies were performed in different patient populations. In NeoTRIP, 49% of patients had locally advanced disease, whereas in Impassion031 and KEY-NOTE-522, approximately 75% had stage II disease, and 25% had stage III desease^53,63^. The patients enrolled in NeoTRIP therefore had higher conventionally defined risk than those in the 2 other phase III trials of neoadjuvant ICIs.

Another relevant difference in the findings collected in NeoTRIP to date is that PD-L1^+^ had a higher probability of pCR than PD-L1 negative tumors, regardless of the use of atezolizumab, as also shown in multivariate analysis. In Impassion031, the PD-L1-positive subgroup had a higher likelihood of pCR in only the control arm of neoadjuvant chemotherapy. However, in the latter trial, the improved antitumor activity associated with the ICI was statistically present independently of PD-L1 status. Very high expression of PD-L1 might possibly reflect high tumor infiltration by immune cells that cooperate with chemotherapy irrespective of atezolizumab, whereas at lower expression, the antibody may play a different immune-modulatory role^68–70^. The PD-L1 assays and scores used in NeoTRIP and Impassion031 make reconciling the discrepant findings in the 2 studies.

Another difference among trials is that Impassion031 and KEYNOTE-522 used sequential neoadjuvant regimens including an anthracycline combination, whereas NeoTRIP used an anthracycline-free neoadjuvant regimen. Anthracyclines induce potent immunogenic cell death that may increase the likelihood of response of PD-L1 negative tumors^63^, in which the immune priming phase is dysfunctional^71,72^. However, the hypothesis of a possible role of the immune-modulatory qualities of anthracyclines on the probability of pCR is not supported by the reported findings of GeparNUEVO^71^. In that phase II neoadjuvant trial, the addition of the anti-PD-L1 durvalumab did not significantly increase pCR despite the presence of 4 cycles of anthracyclines in the sequential chemotherapy regimen^72^. The apparent discrepancy among trials in terms of antitumor activity with neoadjuvant ICIs can only be reconciled by the expression of PD-L1, which was assessed with different reagents and scoring systems among studies, and was used to stratify patients in the NeoTRIP trial^73^. Given that TILs are positively correlated with the likelihood of pCR, the imbalance/instability might have influenced the results. A further need for caution regarding the relevance of pCR has been indicated by recent reports showing dramatically improved EFS with neoadjuvant ICIs in KEYNOTE-522, including in patients who did not achieve pCR^66^ and in GeparNUEVO despite the lack of a significantly higher pCR rate with durvalumab^72^. The reports support that pCR may not be the most appropriate surrogate endpoint to measure the role of ICIs in neoadjuvant settings for TNBC.

In NeoTRIP, administration of atezolizumab was feasible without the emergence of limiting immune toxicity, which was most frequently characterized by effects on thyroid function, as already known and reported for the drug in women (patients) with BC as well as other indications. Without data on long term efficacy, an appropriate balance between tolerability and benefit cannot be assessed.

In summary, the present analysis of NeoTRIP shows that atezolizumab with nab-paclitaxel and carboplatin is feasible but does not improve the antitumor activity of chemotherapy, measured as cCR (clinical complete responce) and pCR (pathological complete responce) in women with TNBC. However, the primary endpoint of NeoTRIP is EFS, and the lack of pCR improvement may be misleading regarding the effects of atezolizumab on efficacy and survival in high risk TNBC^69^.

The introduction of ICIs has changed the landscape of treatment options in an ever-growing number of oncology indications, and the field is rapidly moving toward setting new standards for TNBC therapy in early disease^74^, as has already been accomplished in metastatic setting.

The current report of NeoTRIP contributes to the ongoing understanding of the use of ICIs in TNBC, and underscores the need for dependable predictors of activity and efficacy of ICI. Follow-up, analysis, and molecular characterization of the vast collection of tumor and blood specimens in NeoTRIP are ongoing, and will provide additional contributions.

## New combinatorial strategy

Zhang et al.^75^ have examined the efficacy and safety of a sequential combination of chemotherapy (anthracycline-or taxane-based regimens) and autologous cytokine-induced killer (CIK) cell immunotherapy in patients with TNBC. In the CIK group, the DFS and OS intervals were significantly longer than those in the control group (DFS: *P* = 0.047; OS: *P* = 0.007). The strategy of CIK cell therapy after adjuvant chemotherapy may decrease recurrence and metastases postoperatively in TNBC, thereby prolonging the overall survival time with minimal adverse effects. Therefore, CIK cell immunotherapy may be a potential new strategy for systemic adjuvant therapy after surgery for patients with TNBC in the near future.

## Safety

The safety profile of the atezolizumab-paclitaxel combination in the IMpassion 131 trial was consistent with the effects in other similar trials. The incidence of atezolizumab-treated patients with hypothyroidism was identical in IMpassion 130 and IMpassion 131 (14%, any grade).

The most common adverse events (>25%) were alopecia, anemia, peripheral neuropathy, diarrhea, fatigue, and nausea, all of which were more common with atezolizumab-containing therapy than paclitaxel or nab-paclitaxel ^+^ placebo^59,60^.

## Conclusions

The introduction of ICIs has changed the landscape of treatment options in an ever-growing number of oncology indications, and the field is rapidly moving toward setting new standards of therapy for TNBC in early stages, as has already been accomplished in metastatic settings^61^. The current report of NeoTRIP contributes to the ongoing understanding of the use of ICIs in TNBC and underscores the need for dependable predictors of activity and efficacy of ICI. The follow-up of the study and the analysis and molecular characterization of a vast collection of tumor and blood specimens in NeoTRIP is ongoing, and will provide additional contributions.

TNBC, unlike HR^+^ cancer, is a subgroup characterized by poor prognosis, rapid progression to metastatic stage, and rapid onset of resistance to chemotherapy after the initial response. TNBC represents a specific area of medical need in which new therapeutic approaches warrant appropriate testing. The expression of immune regulatory checkpoints, such as PD-1 and its ligand B7-H1 (or PD-L1), negatively affect the results of treatments. A subset of patients have been found to have an ongoing immune response within the tumor micro environment, and PD-L1 expression is an adaptive method of tumor resistance to TILs, which in turn are needed for the response to chemotherapy. Overall, data suggest a role of immune regulation of response to chemotherapy and support the concept that immune checkpoint blockade may favor the achievement of durable response through immune mechanisms themselves and in combination with classical chemotherapy.

Relevant differences in the findings in the NeoTRIP trial are that PD-L1^+^ was associated with a higher probability of pCR than PD-L1 negative tumors, regardless of the use of atezolizumab. In other trials (Impassion031) the PD-L1 positive subgroup had a higher likelihood of pCR in the control arm receiving neoadjuvant chemotherapy only. However, in the latter trial, the improved antitumor activity associated with the ICI was statistically independent of PD-L1 status.

Very high expression of PD-L1 might reflect high tumor infiltration by immune cells, which cooperate with chemotherapy regardless of atezolizumab, whereas at lower expression, the antibody may play a different immune-modulatory role.

The introduction of ICIs has changed the landscape of treatment options in increasing oncology indications, and the field is rapidly moving toward setting new standards of therapy for TNBC and HER2^+^ early disease, as has already been accomplished in metastatic settings. However, not all diagnostic markers approved for cancer immunotherapy, including PD-L1 expression, are always predictive of the response to ICI. Some patients with BC (particularly HR^+^ BC) do not benefit from therapy with these drugs. Therefore, biomarkers must be identified for better stratification of PD-L1 positive patients with BC potentially sensitive to ICI. In the past 2 years,^76^ the new immunosuppressive immunomarker leukemia inhibitory factor (LIF) has been identified. High levels of LIF are associated with shorter survival of patients after anti PD-L1 therapy. In contrast, low levels of LIF are associated with high levels of TILs and high density of CD8^+^ T-lymphocytes. The presence of TILs is highly predictive of improved outcomes in cancer patients treated with ICI. Therefore, targeting the LIF axis may provide a reliable future approach to improve the efficacy of ICI therapy in patients with PD-L1^+^ BC.
